# Hepatitis B Viral Protein HBx: Roles in Viral Replication and Hepatocarcinogenesis

**DOI:** 10.3390/v16091361

**Published:** 2024-08-26

**Authors:** Dong Li, Yassir Hamadalnil, Thomas Tu

**Affiliations:** 1 The Westmead Institute for Medical Research, Faculty of Medicine, The University of Sydney, Westmead, NSW 2145, Australia; doli8277@uni.sydney.edu.au; 2 Faculty of Medicine, Nile University, Khartoum HJW8+2P2, Sudan; aboalyoosr@gmail.com; 3 Centre for Infectious Diseases and Microbiology, Sydney Infectious Diseases Institute, The University of Sydney at Westmead Hospital, Westmead, NSW 2145, Australia

**Keywords:** hepadnavirus, HBV DNA integration, cccDNA, DBB1, hepatocellular carcinoma, HCC, cancer evolution, transcriptional regulation

## Abstract

Hepatitis B virus (HBV) infection remains a major public health concern worldwide, with approximately 296 million individuals chronically infected. The HBV-encoded X protein (HBx) is a regulatory protein of 17 kDa, reportedly responsible for a broad range of functions, including viral replication and oncogenic processes. In this review, we summarize the state of knowledge on the mechanisms underlying HBx functions in viral replication, the antiviral effect of therapeutics directed against HBx, and the role of HBx in liver cancer development (including a hypothetical model of hepatocarcinogenesis). We conclude by highlighting major unanswered questions in the field and the implications of their answers.

## 1. Introduction

Infection with the hepatitis B virus (HBV) is one of the most common bloodborne diseases, having infected approximately one third of the world’s population and chronically affecting 296 million people [1]. Viral persistence and the chronic host immune responses against infected cells drive ongoing liver injury, which can result in cirrhosis and liver cancer—disease states that kill ~1 million people per year [2,3]. One of the most long-standing mysteries surrounding HBV is the function of the hepatitis B X protein (HBx), a small trans-activator that has a disordered structure and multiple reported roles in viral replication and cancer development [4]. Here, we review the current state of the art on the roles of functions of this enigmatic protein and potential therapeutics to inhibit it. 

## 2. Hepadnavirus Characteristics

HBV is a member of the Hepadnaviridae family, which is composed of two major groups: avihepadnaviruses and orthohepadnaviruses, infecting birds and mammals, respectively. All hepadnaviruses are small, enveloped DNA viruses with partially double-stranded circular genomes of ~3.2 kilobase pairs (kbp) in length [5]. The virions of hepadnaviruses are spherical and ~40 nm in diameter, consisting of a lipid envelope studded with viral surface antigens surrounding a nucleocapsid composed of viral core antigen [6,7]. The circular genome is fastened by cohesive overlaps between the 5′ ends of the two DNA strands, with a covalently linked viral polymerase at the 5′ end of its minus strand [5,8,9].

With respect to genomic structure, all hepadnaviruses have at least three partially overlapping open reading frames (ORFs: P, C and S) that encode several proteins [10]. The P ORF encodes HBV polymerase that is essential for viral replication, particularly the packaging of pre-genomic RNA and its reverse transcription [11,12]. The PreC/C ORF encodes HBcAg, which makes up the viral capsid, and a secreted e antigen (HBeAg) that has potential immunomodulatory functions [13,14]. The S ORF encodes two (avihepadnaviruses) or three (orthohepadnaviruses) surface antigen (HBsAg) isotypes, which decorate the exterior of the virion and mediate entry into hepatocytes [6].

## 3. Hepadnavirus Life Cycle

**Figure 1:**
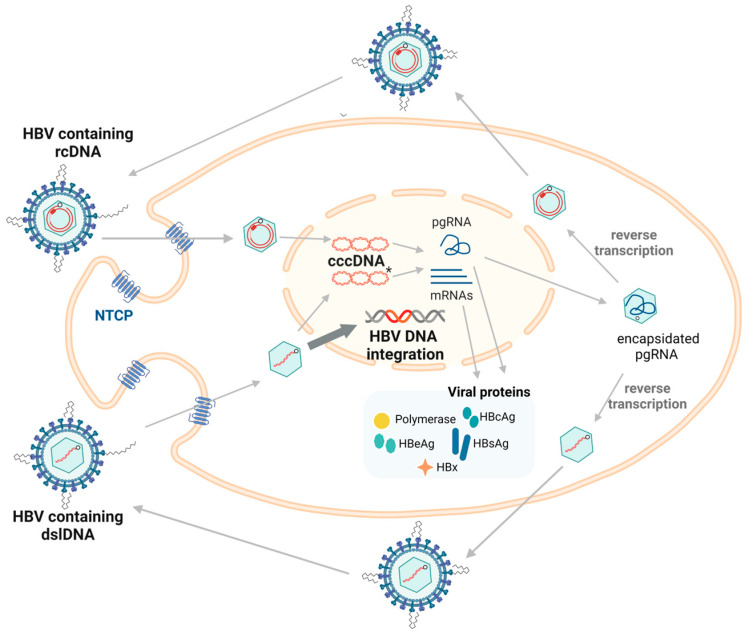
The replication cycle of HBV. The HBV enters the hepatocyte via endocytosis using the cell surface receptor (NTCP). The capsid containing relaxed circular DNA (rcDNA) is transported to the nucleus where it undergoes repair to form cccDNA, which is the main transcriptional template for both pgRNA and mRNAs that are translated into viral proteins. HBcAg forms the nucleocapsid surrounding pgRNA and initiates reverse transcription to re-form viral DNA. Most of the time, rcDNA is formed, with double-stranded linear DNA (dslDNA) alternatively produced as a by-product (5–10% of synthesized virions) [22]. The mature nucleocapsids containing viral DNA are enveloped and secreted as virions. dslDNA can integrate into the host genome at double stranded DNA breaks (highlighted using bold arrow). dslDNA can also form cccDNA but cannot code for functional rcDNA due to an additional 16nt insertion [23]. Figure adapted from [23] and generated in BioRender (https://biorender.com/, accessed on 20 August 2024).

Hepadnaviruses are liver-tropic and mainly infect the hepatocytes (Figure 1). Virions first attach to cell surfaces with low affinity via binding of heparan sulfate proteoglycans. Subsequently, the large envelope protein binds with high affinity to hepatocyte specific receptors (NTCP in the case of human HBV [15], still unknown in many other hepadnaviruses). Following endocytosis and removal of the viral envelope and nucleocapsid, the viral capsid containing the relaxed circular DNA (rcDNA) is transported to the nucleus [16,17], where the genome undergoes repair by host DNA repair enzymes and forms covalently closed circular DNA (cccDNA) [18]. Using cccDNA as the template, viral RNAs are transcribed (among which the HBx transcript is amongst the first in infections by orthohepadnaviruses [19]) and exported into the cytoplasm where they are translated to form the viral proteins. The replicative pre-genomic RNA (pgRNA) is packaged by core protein, forming the viral nucleocapsid [11,12]. The viral DNA-containing nucleocapsids are enveloped at the endoplasmic reticulum and secreted as virions [13]. In avihepadnaviruses, nucleocapsids can also be recycled to the nucleus and contribute to the cccDNA pool of an infected cell [5,8]. In orthohepadnaviruses, this recycling pathway does not appear to be as active, leading to lower numbers of cccDNA molecules per cell (1–10 cccDNA copies per cell, compared to >50 copies in avihepadnavirus infection) [20,21]. 

Besides this replicative cycle, a minority of nucleocapsids contain double stranded linear DNA (dslDNA) rather than rcDNA. These are formed as a by-product during reverse transcription of the pgRNA, which can be released as defective virions, infect surrounding cells, and integrate into the host genome at the site of cellular double-stranded DNA breaks [24,25,26,27]. Integration events are relatively rare, occurring in ~1 per 10,000 infected cells [26,28,29]. Despite their general rarity, integrated forms are strongly associated with liver cancer: ~90% of HBV-associated liver cancers contain HBV DNA integrations [30,31,32,33,34]. 

**Figure 2:**
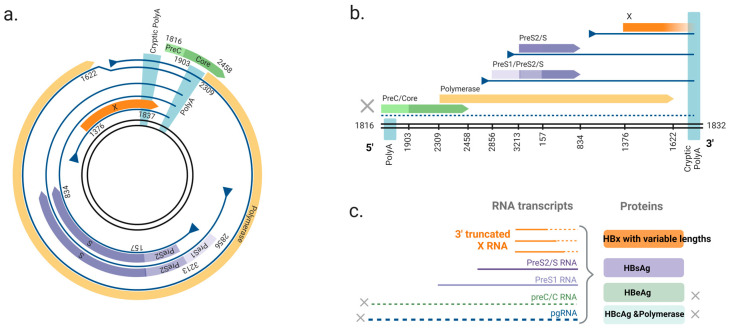
The ORFs of two forms of HBV DNA and derived viral RNAs. (**a**) The circular HBV DNA. The HBV genome consists of four overlapping ORFs: polymerase, pre-core/core, surface and X. The viral mRNAs expressed from circular DNA terminate at a polyadenylation signal (polyA) located in the core ORF. (**b**) dslDNA form HBV DNA. The canonical polyA is located at the 5′ end and mRNAs instead terminate at a non-canonical cryptic polyA. The structural arrangement of the viral genome leads to the separation of pre-c/c and polymerase ORFs from the native promoter (core promoter), leading to loss of mRNA expression (dashed line). The X ORF is truncated at its C-terminus. (**c**) Viral transcripts from the dslDNA and the viral proteins. Pre-c/c RNA (encoding HBeAg) and pgRNA (encoding HBcAg and polymerase) are not transcribed due to the missing 5′ promoter. 3′ end truncated X RNA transcripts are produced from the dslDNA and transcribed to HBx protein with variable lengths. HBV DNA genome: the double black lines; ORFs: colored arrows (green = pre-c/c, yellow = polymerase, purple = surface, orange = x); transcripts: blue lines; promoters: blue triangles. Nucleotide numbering of ORFs is shown as per GenBank Accession #AB241115. Figure adapted from [23] and generated in BioRender (https://biorender.com/, accessed on 20 August 2024).

Integrations are amplified when cells with integrations undergo mitosis [30,31,32,35]. This allows HBV DNA integrations to persist and become the major source of HBV antigens in later phases of chronic infection [33,36,37]. Although HBV DNA integration normally fails to transcribe pre-core mRNA and pgRNA due to the loss of the upstream basal core promoter, the promoters of S and X ORF are intact, allowing the synthesis of functional HBsAg and C-terminal truncated HBx proteins [36,38,39] (Figure 2).

## 4. HBx 

A key difference between avihepadnaviruses and orthohepadnaviruses is the presence of HBx ORF (only in the latter), which encodes the HBx protein and appears to be a function acquired by orthohepadnaviruses to adapt to mammalian hosts during evolution [40].

**Figure 3:**
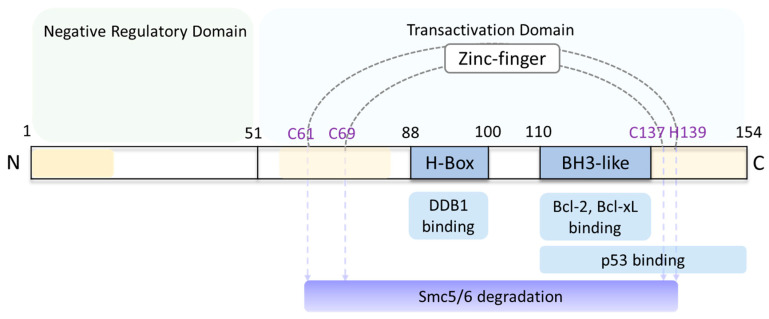
Scheme of HBx protein functional domains. The 154-amino acid (aa) HBx has a negative regulatory domain located at the N-terminus (aa1–50) that is predicted to be predominantly unstructured, and a transactivation domain located at the C-terminus (aa51–154). H-box motif (aa88–100) binds DDB1 and CCCH (C61, C69, C137 and H139) zinc finger motifs, both of which target the Smc5/6 complex for degradation [41,49]. BH3-like motif (aa110–135) binds with Bcl protein family, promoting HBV replication and cytotoxicity [48,50]. p53 binding motif (aa111–154) represses p53-mediated apoptosis [52]. Three conserved regions (aa1–20, aa58–84 and aa120–154) are colored yellow. The figure is adapted from [53].

HBx is a small 154 amino acid (aa) 17 kDa protein. Three conserved regions (aa1–20, aa58–84 and aa120–154) have been identified based on the sequence homologies among eight HBV genotypes [41,42]. The N-terminus functions as a negative regulatory domain (aa1–50), while the C-terminus functions as a transactivation domain (aa51–154) [43,44,45] (Figure 3). The N-terminal domain is predicted to be largely unstructured, suggesting high flexibility for binding with diverse interactors [46,47]. The C-terminal domain is relatively well conserved due to the overlap with several regulatory elements [43,45]. The unstructured domain and the poor solubilization of HBx has led to technical difficulties in elucidating the crystal structure of HBx. While small domains of HBx have been crystalized and have solved structures [48,49,50,51,52], the structure of a fully functional HBx remains unknown. 

HBx is predicted to form homodimers via disulfide bonds and acetylation [54]. Its conformational flexibility may let it develop different secondary structures under specific conditions, such as become folded upon binding to its target molecules (with a wide array of signaling proteins, transcriptional regulators and nucleic acids) [47]. Intracellular localization studies based on HBV patient-derived liver biopsies showed that HBx predominantly accumulates in the cytoplasm when highly expressed, whereas low expression leads to localization primarily in the nucleus [55,56,57]. It has been demonstrated that the localization of HBx to the nucleus is essential for its role in virus replication in vitro and in vivo [13]. Recent discoveries showed that HBx can coordinate a zinc ion (Zn) or an iron-sulfur (Fe–S) cluster through metal-binding cysteine residues located at the C-terminus. This highlights the potential for HBx involvement in metalloprotein-associated functions such as redox regulation, electron transfer, DNA binding and repair mechanisms. This coordination could be important for structural stability, protein–protein interactions, or catalysis, which are key to its various roles in viral replication and the modulation of host cellular processes [49,50,58].

## 5. Role of HBx in HBV Replication

HBx appears to be essential for viral transcription [19,59]. Chen et al. and Zoulim et al. reported that the orthohepandavirus woodchuck hepatitis virus (WHV) bearing mutations in the X gene was unable to infect or initiate infection in susceptible woodchucks, suggesting that the WHV X gene is important for viral replication [59,60]. Indeed, this has also been shown for human HBV in in vitro infection model, as well as in hydrodynamic mouse models [19,61]. Recent studies have begun to clarify the specific roles of HBx in viral replication (Table 1).

### 5.1. HBx and DDB1

HBx appears to overcome cellular restriction factors that inhibit viral transcription. Specific regions of HBx that restore viral transcription have been identified, including a conserved alpha-helical motif (H-box, aa88–100) [51,62,63,79] and a zinc-finger containing transactivating regions (CCCH motif) [41,64] (Figure 3).

In a landmark paper, Decorsiere et al. showed that HBx leads to the degradation of the structural maintenance of the chromosome 5/6 (Smc5/6) complex, which was shown to inhibit the expression of genes from episomal DNA [65]. The Smc5/6 complex bridges DNA molecules through topological entrapment and creates a repressive chromatin structure, therefore silencing viral gene expression [66,80]. Thus, the Smc5/6 complex functions as an HBV restriction factor that blocks viral transcription [65,66].

HBx overcomes this inhibition by binding and recruiting DNA damage-binding protein 1 (DDB1) to the Smc5/6 complex. DDB1 binds Cullin4 (Cul4) as part of an E3 ubiquitin ligase complex that recruits substrate proteins for ubiquitination and degradation [46,51]. By recruiting the DDB1-E3 ligase complex to the Smc5/6 complex, HBx triggers its degradation and therefore allows the transcriptional restriction of cccDNA to be lifted [64,65,66,67]. Indeed, the region of HBx involved in DDB1 binding is the highly conserved H-box domain, suggesting its strong selective advantage and likely role in viral replication of all orthohepadnaviruses.

Other studies support this function of HBx in viral replication. For example, genetic knockdown of Smc5/6 restores the replication of HBx-deficient HBV [66]. A study using clinical samples has shown that the anti-Smc5/6 function can be retained in HBx variants found in patients with hepatocellular carcinoma (HCC) [68]. Cells with either DDB1-binding deficient HBx or Cul4-binding deficient DDB1 exhibited no cccDNA transcription [67]. In the absence of HBx, Smc5/6 anchors cccDNA to nuclear domain 10 (ND10) bodies, nuclear regions of transcriptional repression. This is in contrast to cccDNA in the presence of HBx, which localizes to regions of active transcription [46,67]. Finally, despite the marked diversity of HBx among orthohepadnaviruses (HBx has a percentage identity ranging from 87% compared to orangutan HBV to 30% in Asian grey shrew HBV) [81,82], inhibiting the action of Smc5/6 is a conserved feature in all known members [49,83]. Together, these findings have resulted in a robust model of how HBx controls viral transcription in orthohepadnaviruses. 

### 5.2. HBx and Histone Modification

Other mechanisms by which HBx has been reported to drive HBV transcription include modifying histones. Efficient transcription from cccDNA requires histone binding and transcriptional regulators (e.g., transcription activity is enhanced when the histones are acetylated) [84,85,86]. HBx has been shown to increase HBV transcription through recruiting of histone acetyltransferases such as CREB-binding protein (CBP)/p300 to cccDNA, promoting histone acetylation to maintain active transcription [69]. In the absence of HBx, the viral genome exists in a repressed chromatin state marked by hypoacetylation and histone H3K9 methylation, correlating with the recruitment of histone deacetylases HDAC1 and H3K9 methyltransferase SET domain bifurcated 1 (SETDB1), as well as the recruitment of the heterochromatin protein HP1, and the cccDNA transcribes significantly fewer RNAs [19,69,70,73].

HBx has also been reported to modify the epigenetic landscape of cccDNA and host chromatin through interactions with DNA methyltransferases (DNMTs) to promote transcription [70,87]. HBx binds protein arginine methyltransferase 1 (PRMT1) and the Tudor-domain protein Spindlin-1, blocking the inhibitory activity on HBV transcription [53,71,72]. HBx also can recruit lysine-specific demethylase 1 (LSD1) and lysine methyltransferase Set1A to the viral promoter, which inhibit H3K9me2 demethylation and accumulate activated H3K4me3 to activate viral transcription [74].

### 5.3. HBx and Cell Signaling Pathways

Some studies have reported that HBx can also directly interact with proteins in the cytoplasm to induce cellular changes. HBx has been found to form a complex with proteins such as MEKK1, SEK1, and SAPK/JNK, resulting in prolonged cell survival and maintaining a cellular environment favorable to HBV replication, reviewed in Schollmeier et al. [54], Diao et al. [88], and Agustiningsih et al. [89]. HBx could increase cytosolic calcium levels through modulation of the mitochondrial permeability transition pore, thereby activating the Pyk2/Src and FAK pathways [75,76]. HBx-mediated Ca2+ signaling also facilitates viral core assembly and pgRNA production, both of which are required for virus replication [76,77]. However, cytosolic HBx localization has only been observed in overexpression models. Most of the interactions of HBx with cellular processes have been studied in many different models, often leading to significant overexpression of HBx and outside the context of natural HBV infection. Thus, it remains unclear whether similar manipulations of the cellular machinery by HBx would also occur in the context of an authentic HBV infection [90]. 

## 6. Antiviral Approaches by Therapeutically Targeting HBx Functions 

The transcriptional silencing of cccDNA by targeting HBx may be an effective strategy for antiviral treatments. Small interfering RNA (siRNA) targeting HBx not only down-regulates HBV transcription, but also restores active Smc5/6 [91], as well as decreases CD8+ T cell exhaustion [92]. A small molecule inhibitor nitazoxanide has been shown to interfere with HBx–DDB1 protein interactions and can restore Smc5/6, resulting in silencing of cccDNA transcription [93]. It has also been reported that the NQO1 inhibitor can reduce the expression of HBx in a dose-dependent manner, inhibiting cccDNA transcription via the Smc5/6 pathway [94]. Interferon-induced gene TRIM5γ can promote the ubiquitination degradation of the K95 ubiquitin site of HBx linked to K48, thereby inhibiting HBV replication [95].

In future, therapies may be directed at highly conserved HBx motifs (regulating HBV transcription via altering the histone acetylation) or the H-box motif (the minimal DDB1-binding domain) [64,70,96]. Inhibitors or antibodies against these domains could silence HBV cccDNA and may be used in combination with currently approved HBV reverse transcriptase inhibitors or other drugs in research pipelines.

## 7. HBx in Hepadnavirus-Associated Liver Cancer 

The other major difference between avihepadnaviruses and orthohepadnaviruses is the association with liver cancer (solely with infection with the latter). Hepadnavirus-associated liver cancer has been found in humans [97], woodchucks [98], cats [99], tree shrews [100] and ground squirrels [101], while it is rarely reported in avihepadnavirus-infected birds. Thus, the field has long suspected that HBx contributes to liver pathogenesis and cancer.

The molecular mechanisms regulated by the HBx protein (generally full-length HBx and largely overexpression studies) that drive pathogenesis was reviewed and summarized in a recent comprehensive review [102].

Rather than the cccDNA-encoded HBx being involved in viral replication, it is likely that integrated HBV DNA is the template for the HBx that drives liver cancer. As infected cells undergo mitosis (e.g., during clonal expansion of a cellular clone after acquisition of a driver mutation), they lose cccDNA, resulting in two uninfected daughter cells [103,104]. In contrast, integrated HBV DNA is replicated with the cellular genome and is passed down along the lineage of the clone. Indeed, quantification of specific integrated HBV DNA junctions has been used to measure the level of clonal expansion in infected liver tissues [30,31,32,33]. Moreover, during the dedifferentiation process leading up to liver cancer, hepatocytes lose their susceptibility to HBV infection (due to down-regulation of the receptor NTCP) [105], so no new cccDNA can be formed within the cells. Indeed, this assumption is consistent with the natural history of HBV-associated liver cancer, which occurs in later phases of HBV infection where cccDNA levels are relatively low in the liver (~1/100 to 1/1000 copies per cell) [106] and integrated HBV DNA is the dominant source of viral antigens. Indeed, integrated HBV is also the major form of viral DNA in liver cancers (1–5 copies of integrated HBV DNA vs. 0.00026–1 copies of cccDNA per cell) [107,108,109].

As mentioned above, the integration process leads to terminal deletions of the HBV DNA and subsequent C-terminal truncation of HBx, leading into the cellular sequences. It is unclear whether the HBx derived from integrated HBV DNA may be more pathogenic, but several studies have shown altered phenotypes driven by HBx with C-terminal truncations. C-terminal truncations of HBx decrease HBx steady-state levels, but also impair HBx’s activation of nuclear factor-κB (NF-κB, a master regulator of the cellular stress response) [110]. C-terminal truncations of HBx also abrogate the anti-proliferative and transactivating functions of HBx [111].

On the transcript level, 3′ truncations result in the canonical poly A signal to be cleaved from the HBx RNA. This may result in several types of HBx mRNAs: (1) mRNAs lacking a poly A tail are less stable and more prone to degradation [112]; (2) mRNAs use a poly A signal from the cellular sequence instead, resulting in a chimeric virus–host mRNA; or (3) mRNAs use a cryptic poly A signal in the HBV sequence instead (non-canonical sequence UAUAAA) [113].

Type 1 mRNAs (unstable poly A tail-deficient) are likely to lead to reduced levels of HBx from integrants and therefore mRNAs of this type are unlikely to alter the phenotype of the cell. Type 2 mRNAs (chimeric transcripts) are also unlikely to be a consistent driving factor for liver cancer as integrations are largely randomly distributed across the genome rather than targeting specific oncogenes or tumor suppressor genes. This reduces the likelihood of disrupting or activating genes directly that might promote carcinogenesis. Type 3 mRNAs (cryptic poly A signal) have been investigated as drivers of cancer and their proposed roles will be summarized in the rest of this section (Table 2). 

### 7.1. HBx and Smc5/6 Complex Disruption

Truncated HBx proteins encoded by Type 3 mRNAs would retain an intact H-box motif and therefore are likely to still bind to DDB1 [64,96], thereby promoting the degradation of Smc5/6 complexes. In addition to silencing transcription from episomal DNA, Smc5/6 complexes are crucial for chromosomal stability [65,66] through several mechanisms: (1) coordination of DNA repair through post-translational modification of host repair proteins to establish DNA-damage-dependent cohesion [125]; (2) stabilization of stalled DNA replication forks, preventing their collapse and allowing proper resumption of DNA synthesis [126,127]; and (3) maintenance of telomeres, the protective ends of chromosomes [128].

Indeed, HBx has been shown to drive DNA damage and genomic instability through Smc5/6 complex degradation in both HBV in vitro and in vivo models [125,129]. HBx has been shown to cause impaired homologous recombination (HR) repair of DNA double-strand breaks [129]. Consequently, DNA damage accumulates within the cell, leading to genomic instability [66,78]. Studies have reported that DNA damage accumulates in the liver tissue of HBV-infected humanized chimeric mice, HBx-transgenic mice and human tissues [129,130]. HBx suppressed the HR repair of DNA double-strand breaks, including that induced by a CRISPR-Cas9 system, which was rescued by restoring the Smc5/6 complex [129]. Smc5/6 degradation may also affect cells to genetic errors under conditions of DNA damage (induced by necroinflammation in chronic hepatitis B), and reduced expression of the NSMCE2 subunit, which is associated with increased cancer incidence in mice [131].

HBx-DBB1 also disrupts the function of transcription factor IIH (TFIIH), an essential component of the DNA repair pathway, making cells more sensitive to ultraviolet light and decreasing their ability to repair their DNA, potentially promoting carcinogenesis [44]. HBx-DDB1 may cause alterations in the function of transcription factor p53, which maintains genomic stability by regulating the cell cycle and promoting DNA repair in response to DNA damage [124]. This may induce cell apoptosis and inhibits nucleotide excision repair, thereby compromising genome integrity [124,132].

### 7.2. HBx and Cancer-Related Signaling Pathways

The intracellular concentration of HBx likely increases with additional integration events following each bout of hepatitis and regeneration, resulting in outgrowth of HBx-positive hepatocytes. The sustained high levels of HBx block tumor necrosis factor-α (TNFα) and fatty acid synthase (FAS)-mediated apoptosis by activation of NF κB, thus infected hepatocytes survive immune-mediated damage and resistance to apoptosis [133].

Altered cellular signaling pathways play a critical role in the progression of HBV-associated HCC. C-terminal truncated X protein expression can promote hepatocyte proliferation and reprogram cell metabolism by inhibiting thioredoxin-interacting protein (TXNIP) [114]. The accumulated truncated HBx can prevent apoptosis and lead to the progress of stem cell-like characteristics such as self-renewal, tumorigenicity, chemoresistance, and migration, promoting the neoplastic transformation of the hepatocytes [111,115,116,117,118]. 

Furthermore, C-terminal truncated HBx regulates the transcription of Caveolin-1 and stabilizes LRP6 to maintain the activation of β-catenin, promote the progression of HBV-associated HCC [119], and enhance the invasion and metastasis of HCC cells [116,120]. In addition, integrated viral DNA can lead to the persistent expression of mutated and truncated proteins, which is associated with endoplasmic reticulum and mitochondrial stress responses and may increase the risk of HCC [121,134]. 

C-terminal truncated HBx is associated with increased reactive oxygen species production and mitochondrial DNA damage, marked by 8-oxoguanine formation, suggesting its role in oxidative stress-induced tumor metastasis [122]. Furthermore, in farnesoid X receptor (FXR)-deficient HCC, C-terminal truncated HBx exacerbates oncogenesis by disrupting cell cycle regulation and glucose metabolism [123]. These findings underscore the importance of targeting metabolic disruptions for therapeutic interventions in HBV-associated HCC.

## 8. A Hypothetical Model of HBx-Mediated Carcinogenesis

While many studies have shown that overexpression of HBx can drive multiple oncogenic pathways, it is important to consider the context within which liver cancer develops and the template from which HBx is expressed during this development. 

Given the asymptomatic nature of liver cancer (and therefore its lack of an impact on spreading the virus infection), the induction of HCC is not likely a feature that is selected for in HBV strains. That is to say, liver cancer induction is likely an “unintended” consequence of the pro-viral functions of HBx.

**Figure 4:**
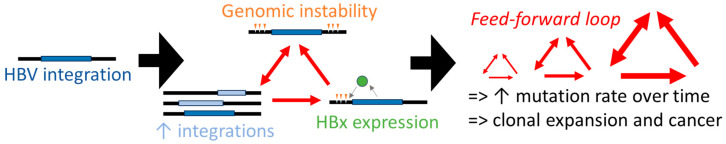
A hypothetical model where cancer development is accelerated by integrated HBV DNA. Genomic instability is driven by virus integrations, which in turn lead to more integrations through the promotion of more double-stranded DNA breaks in the host chromosome (cells with HBV integrations can express functional HBx and may be more susceptible to additional integrations), promoting a self-amplifying process (red arrows). The resultant exponential increasing genomic instability then fuels the accelerated acquisition of cancer driver mutations.

Using the discoveries described in this review, we have developed a hypothetical model with feed-forward loops (Figure 4). We propose that virus integrations drive genomic instability, which in turn may lead to more integrations through the promotion of more double-stranded DNA breaks in the host chromosome. This promotes a self-amplifying process and the resultant exponential increasing genomic instability may fuel the accelerated acquisition of cancer driver mutations.

## 9. Unanswered Questions in HBx Research

Despite recent progress in understanding HBx and its role in both viral replication and carcinogenesis, many unknowns remain in the field. 

### 9.1. What Is the Structure of HBx in Various Cellular Contexts?

Structure determines the function of a protein. Given the high flexibility of its N-terminus, it is likely that HBx takes various forms with different interacting partners. Novel advances in experimental structural analysis (e.g., cryo-electron microscopy) [135] and in silico modeling (e.g., Alphafold 3) [136] may elucidate this. Solving the structures of HBx with key interactors could open up the field of HBx-targeting antiviral therapeutics.

That it is a metalloprotein capable of coordinating a Zn or an Fe–S cluster adds a new dimension to our understanding of its molecular functions [49,50,58]. Fe–S clusters are essential for a range of cellular activities, including enzymatic reactions, regulatory functions, and protection against oxidative damage. For viruses, which rely on the host machinery for replication, the incorporation of such metallocofactors may be vital for altering host cellular pathways, possibly assisting in viral replication, modulation of immune responses, or influencing cellular redox states. Future studies exploring the structural biology of HBx, including how it coordinates these metal ions and the impact on its interactions with host proteins, could lead to novel therapeutic targets, particularly in the context of inhibiting these metal-binding activities to prevent HBV-related liver diseases.

### 9.2. Is Transcriptional Regulation of Integrated HBV DNA Genomes Independent of HBx?

Given that Smc5/6 theoretically only regulates episomal DNA, it is possible that transcription of integrated HBV DNA is not under the restriction activity of Smc5/6. Thus, HBx from integrated HBV DNA may be a mechanism by which transcriptionally silenced cccDNA can be rescued to reactivate an inactive infection. 

Indeed, given the conservation of DDB1 interacting domains, truncated HBx encoded by integrated HBV DNA likely retains the function of maintaining active transcription of cccDNA [137]. If this is shown, then it may suggest that HBx inhibitors may be an additional mechanism by which to prevent HBV reactivation. 

### 9.3. What Are the Most Appropriate Models to Test HBx Function and Anti-HBx Therapies?

Over-expression of HBx can cause aberrant localization and epiphenomena in vitro. Several models have been utilized to test HBx function and anti-HBx therapies, with specific advantages and limitations. The in vitro NTCP-dependent HBV infection system is widely accepted as a robust model for studying the role of HBx in viral replication within a physiologically relevant setting (such as HepG2-NTCP, HepaRG, other engineered cells, or primary human hepatocytes) [90]. However, it is still unclear if there is an optimal model in which to study the roles of HBx in genomic instability and carcinogenic processes. 

### 9.4. Can Anti-HBx Therapies Prevent or Reduce HBV-Associated Liver Cancer?

By transcriptionally silencing (e.g., using siRNA or epigenetic silencing), mutating (e.g., using CRISPR), or inhibiting the function (e.g., via small molecule inhibitors) of HBx, studies have shown effects on HBx-dependent replication and to some extent DNA damage. However, it is still unclear whether these approaches could induce a reduction of HBV-associated liver cancer, independent of their antiviral effects. Indeed, there is not yet an optimal preclinical model to test this question. 

## 10. Conclusions

HBx has various biological functions and may need this flexibility to mediate its function(s) in the different cellular environments encountered during a decades-long chronic HBV infection. Given the clinical importance of chronic HBV infection, the central role of HBx in HBV replication and in the occurrence and progression of HBV-associated HCC, antiviral strategies targeting HBx may be promising clinical treatment strategies. 

## Figures and Tables

**Table 1:** Roles of HBx in Viral Replication.

	HBx Activity	Experimental Models	Year	References
HBx and DDB1	Interferes with cell viability	In vitro primary REFs and HeLa cells transfected with HBx	2001	Lin-Marq et al. [62]
	Stimulates viral genome replication distinct from leading to cell death	In vitro HepG2 and Huh-7 cells transfected with HBx	2005	Leupin et al. [63]
	Anchors viral hijackers and substrate receptors to the Cul4–DDB1 ubiquitin ligase machinery through a promiscuous α-helical motif	In vitro transfection experiments with HeLa and HepG2 cells	2010	Li et al. [51]
	HBx-DDB1 is required for maximal HBV replication	In vitro HepG2 cells transfected with pHBV1.3 and point mutant HBx; in vivo HBx-transgenic mice	2012	Hodgson et al. [64]
	Identified the Smc5/6 complex as a host restriction factor	In vitro transfection of human hepatoma cells and HepaRG cells; in vitro infection of PHH; in vivo infection of human liver chimeric uPA-SCID mice	2016	Decorsière et al. [65]
	Promotes degradation of Smc 5/6 to enhance HBV replication	In vitro wild type and mutant HBx-expressing HepG2, HepAD38, HepG2-NTCP cell lines	2016	Murphy et al. [66]
	Smc5/6 complex restricts HBx-DDB1 when localized to ND10 without inducing an innate immune response	In vitro PHH infection with wild type and mutant HBx HBV; in vivo infection of human liver chimeric uPA-SCID mice	2017	Niu et al. [67]
	HBV replicating in HCC encodes HBx variants with preserved ability to antagonize restriction by Smc5/6	In vitro dHepaRG cells infected with wild type and mutant HBx-expressing HepG2; HeLa cells transfected with HBx	2019	Rivière et al. [68]
HBx and histone modification	HBx is recruited onto the cccDNA with a kinetic paralleling HBV replication	In vitro transiently transfected HepG2 cells with full-length WT and HBx mutant HBV	2009	Belloni et al. [69]
	Epigenetic regulation of HBV transcription from cccDNA	In vitro HBV infection of primary human hepatocytes (PHH) and HepaRG cells with wild type and mutant HBx-expressing HepG2 cells	2011	Lucifora et al. [19]
		In vitro transiently transfected HepG2-NTCP cells with full-length WT and truncated-HBx plasmids	2020	Chong et al. [70]
	Binding to PRMT1/Spindlin-1, blocks the inhibitory activity of PRMT1 on HBV transcription	In vitro transfection of HepG2 cells; in vitro infection of PHH; in vivo mouse model infected with AAV2/8-HBV or AAV2/8-empty virus vector (single tail vein injection)	2013	Benhenda et al. [71]
		In vitro infection of HepaRG cells with HBVwt and HBVX-; HEK293 cell transfection	2014	Ducroux et al. [72]
	HBx relieves chromatin-mediated transcriptional repression of cccDNA involving SETDB1 histone methyltransferase	In vitro HBV infection of PHH and dHepaRG cells with HBVwt or HBVX-	2015	Rivière et al. [73]
	HBx regulates the recruitment of chromatin modifying enzymes(LSD1/Set1A) to an active cccDNA chromatin state	In vitro transient transfection of Huh7 and HepG2 cells	2016	Alarcon et al. [74]
	Epigenetic switch to an H3K4me3-marked active state; a conformational switch may occur in coordination with HBx-DDB1	In vitro HBV infection models in HepG2-NTCP cells	2023	Liu et al. [53]
HBx and cell signaling pathways	HBx targets mitochondrial calcium regulation, thereby activating Pyk2/Src and FAK pathways	In vitro HepG2 cells transfected with full-length HBx	2001, 2007	Bouchard et al., McClain et al. [75,76]
	Enhances HBV core assembly	In vitro HepG2 cells transfected with pHBV1.2x (WT and mutant)	2005	Choi et al. [77]
	A truncated mutant (aa58–140) of HBx retains transactivation function	In vitro transiently transfected HepG2 cells	1996	Kumar et al. [41]
	HBx-deficient HBV genomes are compromised for HBV replication	In vitro transfection of HepG2 cells with pHBV1.2 and an HBx-deficient plasmid; in vivo infection mice model (in hydrodynamic injection)	2009	Keasler et al. [61]
	The stimulation of viral genome replication by HBx is linked to both nuclear and cytoplasmic HBx	in vitro transfection of HepG2 cells with pHBV1.2 Wt or HBx-null construct	2009	Cha et al. [56]
	HBx is indispensable for HBV replication	In vivo infection of human hepatocyte chimeric mice with WT (pHBV1.4) and HBx-def HBV	2010	Tsuge et al. [78]

**Table 2:** A Summary of C-terminal Truncated HBx in Carcinogenesis.

	Pathogenesis of C-Terminal Truncated HBx	Study Design	Year	References
Cancer-related signalling pathways	Abrogates the antiproliferative and transactivation effects of HBx	In vitro HepG2 and MIHA cells transfected with full-length and mutant HBx, HCC patient samples	2008	Ma et al. [111]
	Downregulates TXNIP protein to reprogram glucose metabolism	In vitro HBx-expressing MIHA and LO-2 cell lines; in vivo mice model and HCC patient samples	2021	Zhang et al. [114]
	Induces cancer and stem cell-like properties in HCC cell lines through overexpression	In vitro HCC cells, Huh7 and immortalized normal liver cells MIHA with or without HBx-ΔC mutants stably overexpressed	2016	Ng et al. [115]
	Enhances cell invasiveness and metastasis in HCC by activating MMP10 through C-Jun	In vitro full-length and C-truncated HBx-expressing human hepatoma cells and human HCC samples	2013	Sze et al. [116]
	Regulates tumorigenicity, self-renewal and drug resistance via STAT3/Nanog signaling pathway	In vitro full-length and C-truncated HBx-expressing human hepatoma cells	2017	Ching et al. [117]
	Upregulates transcription of FAS, mediated by 5-lipoxygenase (5-LOX)	In vitro HBx-expressing human hepatoma HepG2 and H7402 cells	2010	Wang et al. [118]
	Activates caveolin-1/LRP6/β-catenin/ FRMD5 axis in promoting hepatocarcinogenesis	In vitro full-length and C-truncated HBx-expressing human hepatoma cells; in vivo mice model; HCC clinical samples	2019	Mao et al. [119]
	Deregulates metastasis suppressors in hepatocellular carcinoma	In vitro full-length and C-truncated HBx-expressing human hepatoma cell lines and HCC samples	2016	Li et al. [120]
	Induces endoplasmic reticulum and mitochondrial stress responses	In vitro transfection in human HCC cells	2016	Montalbano et al. [121]
	Induces oxidative stress-associated tumor metastasis	In vitro transfection in Huh-7, HepG2, and Chang liver cells	2013	Jung et al. [122]
	Disrupts cell cycle regulation and glucose metabolis in FXR-deficient HCC	In vitro transfection with full-length and C-terminal truncated HBx in human Hep3B hepatocellular carcinoma cell line	2023	Wu et al. [123]
Inducing genomic instability	Binds DDB proteins	In vitro wild-type or mutant HBx-expressing HepG2 cell lines	1998	Becker et al. [124]
	Decreases HBx stability and HBV replication, impairs HBx activation of NF-κB and a minimal promoter	In vitro HepG2 cells transfected with full-length HBx and truncation mutants	2011	Lizzano et al. [110]
	Degradation of Smc5/6	In vitro wild type and mutant HBx-expressing HepG2, HepAD38, and HepG2-NTCP cell lines	2016	Murphy et al. [66]
